# Supplementary material for: Evaluating whole-genome sequencing quality metrics for enteric pathogen outbreaks
Source: PeerJ. 2021 Nov 25;9:e12446. doi: 10.7717/peerj.12446 (PMC8627651; doi:10.7717/peerj.12446)
Supplement: Supplemental Information 1 [file peerj-09-12446-s001.docx]

### **Table S1. A. NCBI SRA identifiers for Cluster 1, *Escherichia coli* O26.** https://www.ncbi.nlm.nih.gov/sra

| **Isolate/Strain IDs** | **SRA accessions** | **SAMN IDs** |
| --- | --- | --- |
| 2014C-3572 | SRR1580933 | SAMN02918196 |
| 2015C-4936 | SRR3158300 | SAMN04482314 |
| 2015C-4937 | SRR3158334 | SAMN04482315 |
| 2015C-4938 | SRR3158375 | SAMN04482316 |
| 2015C-4939 | SRR3158386 | SAMN04482317 |
| 2015C-4940 | SRR3158398 | SAMN04482318 |
| 2015C-4942 | SRR3152798 | SAMN04480802 |
| 2015C-4943 | SRR3152799 | SAMN04480803 |
| 2015C-4944 | SRR3158400 | SAMN04482320 |
| 2015C-4945 | SRR3158401 | SAMN04482321 |
| PNUSAE001735 | SRR3040212 | SAMN04296686 |
| PNUSAE001736 | SRR3040271 | SAMN04296687 |
| PNUSAE001737 | SRR3040272 | SAMN04296688 |
| PNUSAE001738 | SRR2982145 | SAMN04287898 |
| PNUSAE001742 | SRR2982148 | SAMN04296690 |
| PNUSAE001743 | SRR2982149 | SAMN04296691 |
| PNUSAE001745 | SRR2982151 | SAMN04287901 |
| PNUSAE001782 | SRR3033400 | SAMN04302098 |
| PNUSAE001783 | SRR3033401 | SAMN04302099 |
| PNUSAE001785 | SRR2982121 | SAMN04304553 |

### **Table S1. B. NCBI SRA identifiers for Cluster 2, *Salmonella enterica* Reading.** www.ncbi.nlm.nih.gov/sra/; see also https://www.cdc.gov/salmonella/reading-07-18/index.html

| **Isolate/Strain IDs** | **SRA accessions** | **SAMN IDs** |
| --- | --- | --- |
| CVM N17S1018 | SRR6740266 | SAMN08541552 |
| CVM N17S1019 | SRR6740267 | SAMN08541553 |
| CVM N17S1020 | SRR6740264 | SAMN08541554 |
| CVM N17S1021 | SRR6740265 | SAMN08541555 |
| PNUSAS033882 | SRR10664370 | SAMN08607421 |
| PNUSAS034531 | SRR6791683 | SAMN08622842 |
| PNUSAS034535 | SRR6782574 | SAMN08607422 |
| PNUSAS031958 | SRR6481320 | SAMN08359692 |
| PNUSAS032349 | SRR6491235 | SAMN08381194 |
| PNUSAS036521 | SRR6902806 | SAMN08799899 |
| PNUSAS036910 | SRR6920570 | SAMN08823473 |
| PNUSAS037915 | SRR6967966 | SAMN08899024 |
| APHI_17-9019_S33 | SRR10037041 | SAMN12659052 |
| APHI_17-6335_S26 | SRR10037042 | SAMN12659051 |
| APHI_17-10375_S35 | SRR10037040 | SAMN12659053 |
| APHI_17-10777_S37 | SRR10037039 | SAMN12659054 |
| APHI_17-12383_S27 | SRR10037038 | SAMN12659055 |
| APHI_17-12727_S28 | SRR10037037 | SAMN12659056 |
| APHI_17-12897_S29 | SRR10037036 | SAMN12659057 |
| APHI_17-13313_S30 | SRR10037035 | SAMN12659058 |
| APHI_18-154_S31 | SRR10037034 | SAMN12659059 |

### **Table S1. C. NCBI SRA identifiers for Cluster 3, *Salmonella enterica* Pomona.** www.ncbi.nlm.nih.gov/sra/

###

| **Isolate/Strain IDs** | **SRA accessions** | **SAMN IDs** |
| --- | --- | --- |
| 2015EL-1655A | SRR5224253 | SAMN06290551 |
| 2015EL-1656A | SRR5227853 | SAMN06294328 |
| 2015EL-1657B | SRR3315955 | SAMN04592576 |
| 2016K-0057 | SRR5224252 | SAMN06290550 |
| 2016K-0474 | SRR3743957 | SAMN05360273 |
| 2016K-0488 | SRR3708002 | SAMN05277823 |
| 2016K-0489 | SRR3708000 | SAMN05277821 |
| 2016K-0490 | SRR3708001 | SAMN05277822 |
| PNUSAS002005 | SRR3499816 | SAMN04958840 |
| PNUSAS002454 | SRR3676330 | SAMN05192652 |
| PNUSAS002455 | SRR3667832 | SAMN05192653 |
| PNUSAS002458 | SRR3713614 | SAMN05232281 |
| PNUSAS002459 | SRR3667944 | SAMN05199856 |
| PNUSAS002460 | SRR3667946 | SAMN05199858 |
| PNUSAS002461 | SRR3732332 | SAMN05263637 |
| S15-11952 | SRR10689496 | SAMN13561740 |
| S15-11953 | SRR10689495 | SAMN13561741 |
| SpomonaCTAB_S5 | SRR10689544 | SAMN13562125 |
| SpomonaCTAB_S6 | SRR10689543 | SAMN13562126 |
| PNUSAS002456 | SRR6967966 | SAMN05360257 |
| PNUSAS002457 | SRR3732275 | SAMN05275338 |
| PNUSAS003161 | SRR4032929 | SAMN05509782 |
| PNUSAS003162 | SRR4046870 | SAMN05582478 |
| PNUSAS003164 | SRR4047000 | SAMN05570513 |
| PNUSAS003166 | SRR4047002 | SAMN05570514 |

### **Table S1. D. NCBI SRA identifiers for Cluster 4, *Shigella sonnei*.** www.ncbi.nlm.nih.gov/sra/

| **Isolate/Strain IDs** | **SRA accessions** | **SAMN IDs** |
| --- | --- | --- |
| PNUSAE012367 | SRR6799227 | SAMN08631036 |
| PNUSAE012367 | SRR6799227 | SAMN08631036 |
| PNUSAE012804 | SRR6927293 | SAMN08832946 |
| PNUSAE013040 | SRR7013798 | SAMN08941729 |
| PNUSAE013219 | SRR7086621 | SAMN09005847 |
| PNUSAE013219 | SRR7086621 | SAMN09005847 |
| PNUSAE013260 | SRR7074902 | SAMN08996077 |
| PNUSAE013833 | SRR7133516 | SAMN09092962 |
| PNUSAE013900 | SRR7268989 | SAMN09374563 |
| PNUSAE014208 | SRR7233661 | SAMN09281177 |
| PNUSAE014626 | SRR7346178 | SAMN09428956 |
| PNUSAE014627 | SRR10664274 | SAMN13536121 |
| PNUSAE014629 | SRR7346174 | SAMN09428953 |
| PNUSAE014630 | SRR7346171 | SAMN09428952 |
| PNUSAE014631 | SRR7346173 | SAMN09428951 |
| PNUSAE014640 | SRR7346160 | SAMN09428937 |
| PNUSAE014641 | SRR7346165 | SAMN09428936 |
| PNUSAE014642 | SRR7346169 | SAMN09428949 |
| PNUSAE016098 | SRR7620684 | SAMN09740747 |
| PNUSAE016099 | SRR7620685 | SAMN09740744 |
| PNUSAE016261 | SRR7620682 | SAMN09740875 |
