# Supplementary material for: Evaluating whole-genome sequencing quality metrics for enteric pathogen outbreaks
Source: PeerJ. 2021 Nov 25;9:e12446. doi: 10.7717/peerj.12446 (PMC8627651; doi:10.7717/peerj.12446)
Supplement: Supplemental Information 2 [file peerj-09-12446-s002.docx]

**Table S2. Scripts used to estimate quality metrics and downsample WGS data files.**

| Script | Programmatic Dependencies | Source | Parameters |
| --- | --- | --- | --- |
| run_assembly_readMetrics.pl  *for calculating coverage and PHRED scores* | perl-5.16.1-MT | github.com/lskatz/CG-Pipeline | Coverage: -e 5700000 for *E. coli* O26, -e 4591637 for *S. enterica* ser. Reading, -e 4490000 for *S. enterica* ser. Pomona, and -e 4839734 for *Shigella sonnei* |
| countReadsWithAmbig.py | perl/5.22.1, python/3.4, and prinseq v0.20.3 | github.com/darlenewagner/NGS_Multi_Heal | --format integer |
| run_assembly_removeDuplicateReads.pl | perl-5.16.1-MT | github.com/lskatz/CG-Pipeline | --downsample calculated based upon calculation: (desired coverage) ÷ (calculated coverage) |
