# Supplementary material for: Evaluating whole-genome sequencing quality metrics for enteric pathogen outbreaks
Source: PeerJ. 2021 Nov 25;9:e12446. doi: 10.7717/peerj.12446 (PMC8627651; doi:10.7717/peerj.12446)
Supplement: Supplemental Information 3 — All pipelines tested on isolates from Clusters 1 through 4. Ambiguous nucleotide content was evaluated using the script, countReadsWithAmbig.py, for all raw and healed reads. Custom scripts available for download from https://github.com/darlenewagner/NGS_Multi_Heal [file peerj-09-12446-s003.docx]

**Table S3. Scripts used to implement read healing pipelines.** All pipelines tested on isolates from Clusters 1 through 4. Ambiguous nucleotide content was evaluated using the script, countReadsWithAmbig.py, for all raw and healed reads. Custom scripts available for download from https://github.com/darlenewagner/NGS_Multi_Heal

| Healing Pipeline | Programmatic Dependencies† | Parameters |
| --- | --- | --- |
| noNmin100 | Custom script, windowQualPrinseqLite_R1andR2.py  and prinseq v0.20.3 | minimum read length = 100bp, ambiguous nucleotides < 1 |
| fastxOnly-3pr | fastx-toolkit v0.0.13 or higher | R1 trimmed 3 bp from 3’,  R2 trimmed 15 bp from 3’ |
| noNmin100-3pr | Custom scripts, windowQualPrinseqLite_R1andR2.py and fastxQualAdaptTrimmer_R1andR2.py, plus prinseq v0.20.3 and fastx-toolkit v0.0.13 | minimum read length = 100bp, ambiguous nucleotides < 1 R1 trimmed 3 bp from 3’,  R2 trimmed 15 bp from 3’ |
| prinseq | Custom script, windowQualPrinseqLite_R1andR2.py  and prinseq v0.20.3 | trim quality from 3’ < 27 or 28  trim quality window = 40 bp  Minimum read length = 40 bp, ambiguous nucleotides < 2 |
| prinseq-3pr | Custom scripts, windowQualPrinseqLite_R1andR2.py and fastxQualAdaptTrimmer_R1andR2.py, plus prinseq v0.20.3 and fastx-toolkit v0.0.13 | trim quality from 3’ < 27 or 28  trim quality window = 40 bp  Minimum read length = 40 bp, ambiguous nucleotides < 2  R1 trimmed 3 bp from 3’,  R2 trimmed 5 bp from 3’ |
| prinseq-5pr3pr | Custom scripts, windowQualPrinseqLite_R1andR2.py and fastxQualAdaptTrimmer_R1andR2.py, plus prinseq v0.20.3 and fastx-toolkit v0.0.13 | trim quality from 3’ < 27 or 28  trim quality window = 40 bp  Minimum read length = 40 bp, ambiguous nucleotides < 2  R1 trimmed 3 bp from 3’,  R2 trimmed 5 bp from 3’  R1 and R2 trimmed 1 bp from 5’ |
| bayesHammer | SPAdes 3.11 or higher | spades.py --only-error-correction |
| † wrappers for custom pipelines require Python 3.3 and Perl v5.12 or higher, SPAdes requires Python 2.7 or higher | | |
