# Supplementary material for: Evaluating whole-genome sequencing quality metrics for enteric pathogen outbreaks
Source: PeerJ. 2021 Nov 25;9:e12446. doi: 10.7717/peerj.12446 (PMC8627651; doi:10.7717/peerj.12446)
Supplement: Supplemental Information 4 — No coordinate masking employed for S. enterica ser. Reading draft assembly, CVM_N17S1020. [file peerj-09-12446-s004.docx]

**Table S4. Chromosomal coordinates masked for prophages prior to LyveSET mapping.** No coordinate masking employed for *S. enterica* ser. Reading draft assembly, CVM_N17S1020.

| Genome accession and organism | Prophage coodinates predicted by PHASTER |
| --- | --- |
| AP010953 from *E. coli* O26 | 612033-662219 bp, 890186-942734 bp, 1169321-1284526 bp, 1317740-1327580 bp, 1471989-1523689 bp, 1555308-1626428 bp, 1726186-1773541 bp, 1869486-1917861 bp, 2108626-2207722 bp, 2235874-2253815 bp, 2372587-2410970 bp, 2526884-2582048 bp, 2667387-2705554 bp, 2738066-2754369 bp, 2818311-2828830 bp, 3074880-3153988 bp, 3258875-3321036 bp, 3604877-3660534 bp, and 4952573-4966573 bp |
| CP020718 from *S. enterica* ser. Pomona | 54065-87732 bp, 966604-976551 bp, 1007224-1030252 bp, and 2017365-2031830 bp |
| CP022455 from *Shigella sonnei* | 324608-338617 bp, 518382-528361 bp, 861580-867341 bp, 865859-886478 bp, 984325-996082 bp, 1009471-1024781 bp, 1530602-1584508 bp, 1807065-1817270 bp, 1880844-1907790 bp, 1928304-1941935 bp, 4580196-4611985 bp, and 4614585-4630632 bp |
