# Supplementary material for: Evaluating whole-genome sequencing quality metrics for enteric pathogen outbreaks
Source: PeerJ. 2021 Nov 25;9:e12446. doi: 10.7717/peerj.12446 (PMC8627651; doi:10.7717/peerj.12446)
Supplement: Supplemental Information 5 — R1/R2 PHRED quality, median insert lengths, and percentage of Ns in R1 + R2 reads across trimming/healing methods are indicated by range of values. Dunn test post-hoc p-values are given in parenthesis for each healing method’s comparison with raw reads. Significant p-values ( α < 0.05) in boldface. [file peerj-09-12446-s005.docx]

**Table S5. Quality metrics and variation across read trimming/healing methods.**  R1/R2 PHRED quality, median insert lengths, and percentage of Ns in R1 + R2 reads across trimming/healing methods are indicated by range of values. Dunn test post-hoc p-values are given in parenthesis for each healing method’s comparison with raw reads. Significant p-values (α < 0.05) in boldface.

|  |  | ***E. coli* O26 (Cluster 1)** | ***S. enterica* Reading (Cluster 2)** | ***S. enterica* Pomona (Cluster 3)** | ***Shigella sonnei* (Cluster 4)** |
| --- | --- | --- | --- | --- | --- |
| **raw reads range**  (Kruskal-Wallis p-value) | R1 qual. | 33.0 – 36.1 (**2.39 x 10^–9^**) | 32.5 – 35.7 (**3.38 x 10^–15^**) | 32.2 – 36.9 (5.71 x 10^–2^) | 34.2 – 36.8 (**9.499 x 10^–4^**) |
|  | R2 qual. | 26.0 – 33.5 (**3.38 x 10^–11^**) | 27.5 – 32.8 (**2.20 x 10^–16^**) | 26.1 – 35.3 (**8.32 x 10^–4^**) | 30.2 – 35.4 (**9.24 x 10^–11^**) |
|  | Read length | 181.6 – 234.8 (**9.11 x 10^–16^**) | 180.6 – 245.1 (**8.20 x 10^–16^**) | 147.6 – 292.0 (**1.90 x 10^–8^**) | 191.5 – 245.7 (**5.95** **x 10^–4^**) |
|  | Insert length | 161 – 371 (0.858) | 175 – 531 (0.978) | 184 – 556 (0.973) | 212 – 492 (0.826) |
|  | R1 + R2 Ns % | 0.0 – 5.1 (**3.407 x 10^–16^**) | 0.0 – 4.6 (**2.707 x 10^–4^**) | 0.0 – 31.9 (**2.20 x 10^–16^**) | 0.0 –12.2 (**2.20 x 10^–14^**) |
| **fastxOnly-3pr**  (Dunn Test p-value) | R1 qual. | 33.1 – 36.1 (0.501) | 32.8 – 35.3 (0.299) | 32.3 – 36.9 (0.546) | 34.3 – 36.9 (0.584) |
|  | R2 qual. | 26.5 – 33.8 (0.357) | 27.6 – 32.8 (0.437) | 26.8 – 35.6 (0.443) | 30.5 – 35.6 (0.339) |
|  | Insert length | 171 – 372 (0.602) | 176 – 532 (0.517) | 198 – 558 (0.517) | 213 – 493 (0.525) |
|  | R1 + R2 Ns % | 0.0 – 4.7 (0.504) | 0.0 – 4.5 (0.487) | 0.0 – 31.9 (0.532) | 0.0 – 3.2 (0.490) |
| **prinseq**  (Dunn Test p-value) | R1 qual. | 34.4 – 36.7 (**2.33 x 10^–4^**) | 34.5 – 36.7 (**8.94 x 10^–4^**) | 33.9 – 37.2 (8.70 x 10^–2^) | 35.0 – 37.2 (**2.05 x 10^–3^**) |
|  | R2 qual. | 30.8 – 35.0 (**1.36 x 10^–4^**) | 31.4 – 34.6 (**4.05 x 10^–7^**) | 30.8 – 36.2 (**2.09 x 10^–2^**) | 33.0 – 36.2 (**9.85 x 10^–5^**) |
|  | Insert length | 192 – 382 (0.752) | 187 – 539 (0.886) | 229 – 564 (1.00) | 219 – 494 (0.606) |
|  | R1 + R2 Ns % | 0.0 – 3.5 (**4.23 x 10^–4^**) | 0.0 – 3.2 (**3.18 x 10^–2^**) | 0.0 – 1.5 (**1.66 x 10^–6^**) | 0.0 – 3.7 (**3.39 x 10^–4^**) |

| **prinseq-3pr**  (Dunn Test p-value) | R1 qual. | 34.5 – 36.7 (**1.89 x 10^–4^**) | 34.5 – 36.7 (**2.73 x 10^–6^**) | 34.1 – 37.2 (0.136) | 35.0 – 37.2 (**2.85 x 10^–3^**) |
| --- | --- | --- | --- | --- | --- |
|  | R2 qual. | 31.1 – 35.2 (**1.48 x 10^–5^**) | 31.6 – 34.8 (**4.75 x 10^–8^**) | 31.3 – 36.3 (**1.51 x 10^–2^**) | 33.1 – 36.3 (**3.96 x 10^–5^**) |
|  | Insert length | 192 – 383 (0.925) | 187 – 546 (0.979) | 229 – 564 (1.00) | 220 – 494  (0.625) |
|  | R1 + R2 Ns % | 0.0 – 3.4 (**4.03 x 10^–4^**) | 0.0 – 3.2 (**3.35 x 10^–2^**) | 0.0 – 1.5 (**1.91 x 10^–6^**) | 0.0 – 2.3 (**1.97 x 10^–4^**) |
| **prinseq-5pr3pr**  (Dunn Test p-value) | R1 qual. | 34.5 – 36.7 (**2.39 x 10^–4^**) | 34.6 – 36.8 (**1.00 x 10^–6^**) | 34.1 – 37.2 (0.197) | 35.0 – 37.3 (**3.67** **x 10^–3^**) |
|  | R2 qual. | 31.1 – 35.2 (**2.35 x 10^–5^**) | 31.6 – 35.2 (**3.41 x 10^–8^**) | 31.3 – 36.3 (**2.26 x 10^–2^**) | 33.1 – 36.3 (**6.19 x 10^–5^**) |
|  | Insert length | 190 – 380 (0.742) | 183 – 538 (0.506) | 227 – 562 (1.00) | 218 – 492 (0.541) |
|  | R1 + R2 Ns % | 0.0 – 3.4 (**3.68 x 10^–4^**) | 0.0 – 3.1 (**3.56 x 10^–2^**) | 0.0 – 1.4 (**2.30 x 10^–6^**) | 0.0 – 2.3 (**2.85 x 10^–4^**) |
| **bayesHammer**  (Dunn Test p-value) | R1 qual. | 33.1 – 36.1 (0.423) | 32.6 – 35.7 (0.461) | 32.3 – 36.9 (0.505) | 34.2 – 36.9 (0.611) |
|  | R2 qual. | 26.8 – 33.6 (0.440) | 27.6 – 32.8 (0.449) | 26.3 – 35.4 (0.469) | 30.4 – 35.5 (0.460) |
|  | Insert length | 189 – 412 (1.000) | 182 – 540 (1.000) | 227 – 587 (1.000) | 216 – 499 (0.664) |
|  | R1 + R2 Ns % | 0.0 – 1.7 (6.01 x 10^–2^) | 0.0 – 0.6 (0.178) | 0.0 – 32.0 (**2.91 x 10^–4^**) | 0.0 – 6.5 (**8.75 x 10^–3^**) |
