# Supplementary material for: Evaluating whole-genome sequencing quality metrics for enteric pathogen outbreaks
Source: PeerJ. 2021 Nov 25;9:e12446. doi: 10.7717/peerj.12446 (PMC8627651; doi:10.7717/peerj.12446)
Supplement: Supplemental Information 6 — Total proportions of reads mapped to reference genomes and proportions of reads mapping with proper pairing. Dunn test post-hoc p-values are given in parenthesis for each healing method’s comparison with raw reads. Significant p-values ( α < 0.05) are in boldface. [file peerj-09-12446-s006.docx]

**Table S6.** **Variation in Lyve-SET read-mapping** **Quality metrics**. Total proportions of reads mapped to reference genomes and proportions of reads mapping with proper pairing. Dunn test post-hoc p-values are given in parenthesis for each healing method’s comparison with raw reads. Significant p-values (α < 0.05) are in boldface.

|  |  | ***E. coli* O26 (Cluster 1)** | ***S. enterica* Reading (Cluster 2)** | ***S. enterica* Pomona (Cluster 3)** | ***Shigella sonnei* (Cluster 4)** |
| --- | --- | --- | --- | --- | --- |
| **raw reads range** | Total mapped | 53.18% – 76.57% | 54.44% – 92.85% | 41.14% – 95.67% | 58.88% – 82.57% |
|  | Properly paired | 26.57% – 63.64% | 34.44% – 77.36% | 18.26% – 90.69% | 45.75% – 73.05% |
| **nonMin**  (Dunn Test p-value) | Total mapped | 52.60% – 77.84% (0.4709) | 54.08% – 92.69% (0.4949) | 39.56% – 95.68% (0.4319) | 58.91% – 82.65% (0.5403) |
|  | Properly paired | 25.83% – 66.61% (0.4079) | 34.46% – 77.82% (0.4881) | 17.33% – 91.44% (0.4786) | 47.59% – 73.95% (0.3890) |
| **fastxOnly-3pr**  (Dunn Test p-value) | Total mapped | 54.41% – 76.74% (0.4804) | 54.91% – 93.26% (0.4572) | 52.13% – 95.85% (0.4319) | 59.05% – 82.75% (0.5010) |
|  | Properly paired | 40.65% – 71.70% (**3.165 x 10^–4^**) | 45.81% – 87.65% (**4.961 x 10^–3^**) | 17.33% – 91.44% (0.4602) | 56.22% – 80.20% (**1.179 x 10^–4^**) |
| **noNmin100-3pr**  (Dunn Test p-value) | Total mapped | 53.85% – 77.99% (0.4804) | 54.90% – 93.30% (0.43350) | 50.91% – 95.82% (0.44292) | 59.15% – 82.75% (0.552407) |
|  | Properly paired | 39.36% – 74.09% (**7.110 x 10^–4^**) | 45.35% – 87.69% (**4.697 x 10^–3^**) | 34.94% – 93.88% (**2.996 x 10^–2^**) | 56.31% – 80.14% (**1.077 x 10^–4^**) |
| **prinseq**  (Dunn Test p-value) | Total mapped | 67.46% – 81.01% (**2.506 x 10^–5^**) | 64.25% – 98.56% (**1.066 x 10^–2^**) | 86.81% – 96.60% (**3.352 x 10^–8^**) | 61.16% – 84.67% (1.000) |
|  | Properly paired | 49.74% – 71.39% (**2.251 x 10^–3^**) | 48.71% – 91.11% (**1.038 x 10^–2^**) | 66.22% – 93.33% (**4.544 x 10^–3^**) | 49.30% – 77.12% (**8.848 x 10^–2^**) |
|  |  |  |  |  |  |
| **prinseq-5pr3pr**  (Dunn Test p-value) | Total mapped | 67.38% – 80.89% (**2.391 x 10^–5^**) | 64.62% – 98.81% (**1.102 x 10^–2^**) | 86.93% – 96.65% (**8.035 x 10^–9^**) | 61.18% – 84.59% (0.3833) |
|  | Properly paired | 65.61% – 79.42% (**3.990 x 10^–12^**) | 61.71% – 97.79% (**3.524 x 10^–7^**) | 84.79% – 95.73% (**7.016 x 10^–11^**) | 60.43% – 83.83% (**2.334 x 10^–7^**) |
| **prinseq-3pr**  (Dunn Test p-value) | Total mapped | 67.42% – 80.91% (**2.929 x 10^–5^**) | 64.55% – 98.78% (**9.974 x 10^–3^**) | 86.95% – 96.67% (**6.463 x 10^–9^**) | 61.21% – 84.66% (0.6955) |
|  | Properly paired | 65.74% – 79.47% (**3.426 x 10^–12^**) | 61.66% – 97.78% (**1.762 x 10^–7^**) | 84.80% – 95.76% (**7.458 x 10^–11^**) | 60.50% – 83.91% (**3.246 x 10^–7^**) |
| **bayesHammer**  (Dunn Test p-value) | Total mapped | 64.82% – 79.20% (**6.435 x 10^–3^**) | 58.93% – 95.45% (0.1315) | 62.80% – 96.36% (**3.780 x 10^–2^**) | 60.70% – 83.94% (0.2529) |
|  | Properly paired | 43.19% – 67.94% (**5.201 x 10^–2^**) | 40.41% – 84.10% (0.1733) | 36.30% – 92.75% (0.1998) | 47.18% – 75.42% (0.2122) |
