# Supplementary material for: Evaluating whole-genome sequencing quality metrics for enteric pathogen outbreaks
Source: PeerJ. 2021 Nov 25;9:e12446. doi: 10.7717/peerj.12446 (PMC8627651; doi:10.7717/peerj.12446)
Supplement: Supplemental Information 7 [file peerj-09-12446-s007.docx]

### **Table S7. Assembly quality metrics average values for Skesa.**

|  | **Metric** (Kruskal-Wallis p) | Raw Reads | noNmin100-3pr | prinseq | prinseq -5pr3pr | prinseq-3pr | bayesHammer |
| --- | --- | --- | --- | --- | --- | --- | --- |
| Cluster 1 -*E. coli* O26  **Kruskal-Wallis**  **df = 7** | Contigs (0.5501) | 340.6 | 337.4 | 318 | 320 | 317.7 | 303.6 |
|  | N50 (0.629) | 60,545.8 | 64,119.3 | 64,255 | 66,153 | 64,274.0 | 75,147.9 |
|  | Maximum contig (0.5416) | 175,486.1 | 168,105.1 | 189,172 | 195,148 | 191,369.5 | 202,003.1 |
| Cluster 2 -*S. enterica* Reading  df = 7 | Contigs (0.9329) | 194.4 | 185.7 | 140 | 142.4 | 139.5 | 128.3 |
|  | N50 (0.9493) | 117,216.5 | 119,508.4 | 135,941 | 134,227.2 | 130,616.3 | 152,296.7 |
|  | Maximum contig (0.9408) | 321,910.6 | 326,246.5 | 362,803 | 344,451.29 | 345,241.8 | 366,842.1 |
| **Cluster 3- *S. enterica* Pomona**  **df = 7** | Contigs (0.8512) | 49.4 | 48.9 | 43.4 | 42.1 | 42.5 | 42.2 |
|  | N50 (0.8932) | 242,574.9 | 241,382.7 | 264,960.7 | 261,754.3 | 262,526.2 | 275,549.8 |
|  | Maximum contig (0.7062) | 478,739.2 | 490,008.0 | 558,595.7 | 554,206.5 | 518,485 | 555,772.5 |
| **Cluster 4 - *Shigella sonnei***  **df = 7** | Contigs (0.9371) | 391.6 | 390.1 | 388.8 | 390.8 | 389.7 | 386.4 |
|  | N50 (0.9741) | 23,494.1 | 23,515 | 23,615.8 | 23,443.8 | 23,707.1 | 23,761.6 |
|  | Maximum contig (0.9071) | 88,610.4 | 87,767.8 | 87,860.5 | 89,416.9 | 88,876.0 | 88,500.0 |
|  |  |  |  |  |  | |  |
