# Supplementary figures and images for: Evaluating whole-genome sequencing quality metrics for enteric pathogen outbreaks
Source: PeerJ. 2021 Nov 25;9:e12446. doi: 10.7717/peerj.12446 (PMC8627651; doi:10.7717/peerj.12446)

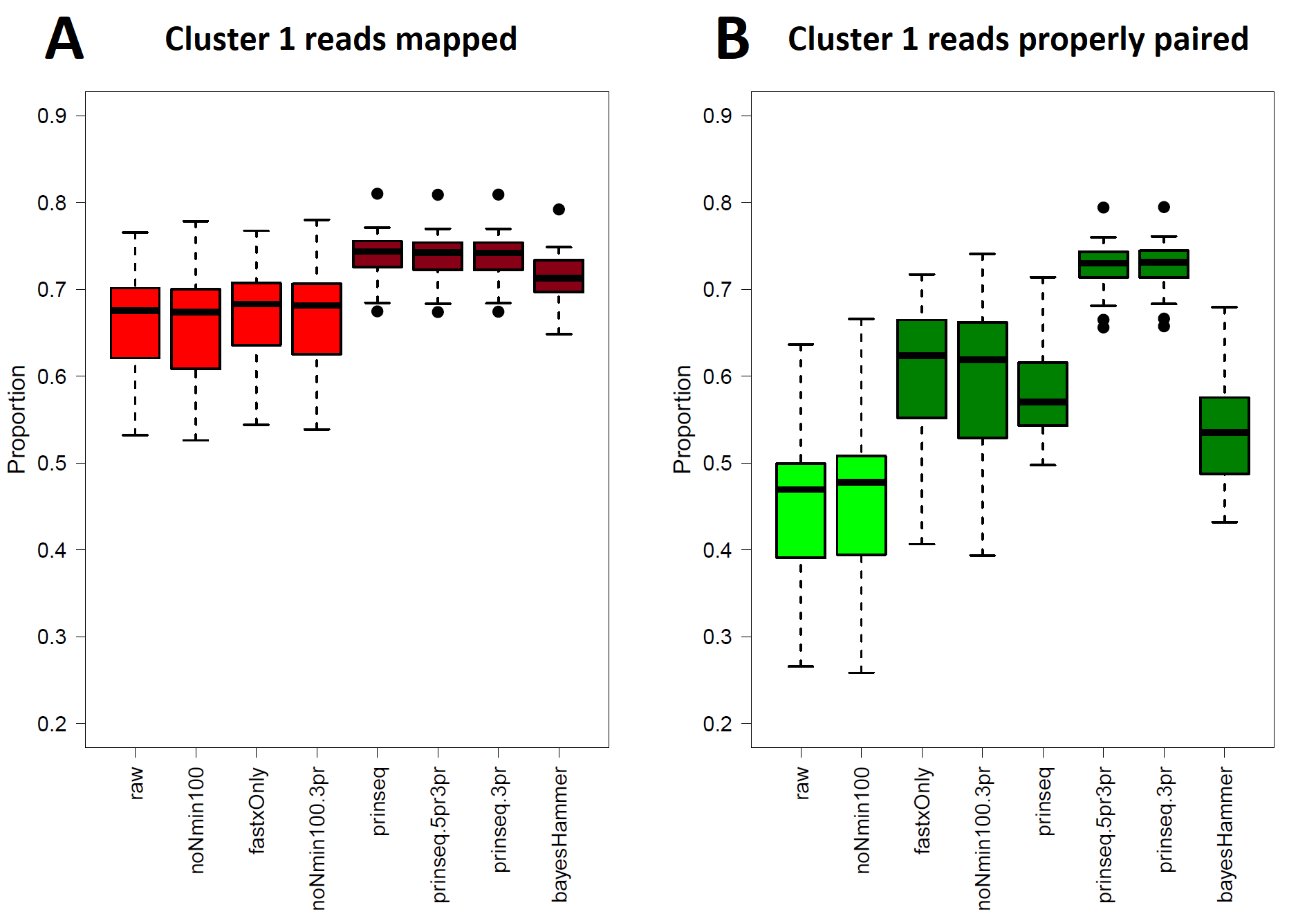

Supplement: Supplemental Information 13 — Total proportions of reads mapped to reference genome, AP010953 (Data S1, column M). Kruskal–Wallis p = 6.203 × 10^−12^ ( df = 7); prinseq, prinseq-5pr3pr, prinseq-3pr, and bayesHammer read mappings differ from raw reads by p < 0.05 under the pairwise comparisons post hoc test (Table S5). (B) Proportions of reads mapping with proper pairing (Data S1, column N) against reference genome AP010953. Kruskal–Wallis p = 2.20 × 10^−16^ ( df = 7); All healed reads except noNmin100 show improved proper paired mapping by p < 0.05 under the pairwise comparison post hoc test. [file peerj-09-12446-s013.png]

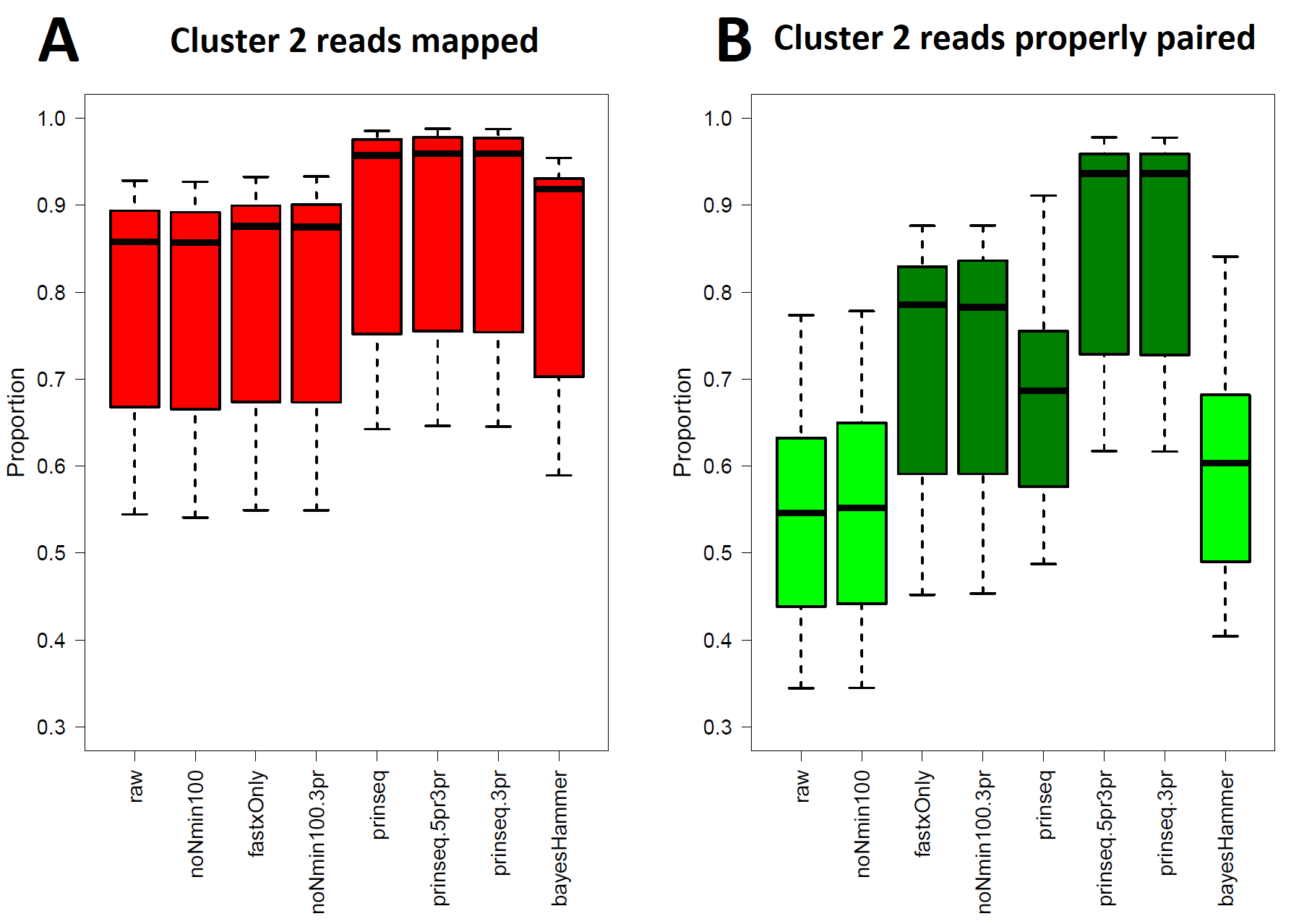

Supplement: Supplemental Information 14 — Total proportions of reads mapped (Data S2, column M) to assembly of strain CVM_N17S1020. Kruskal–Wallis p = 0.000302 ( df = 7) indicates that none of the healing pipelines significantly improve total read mapping. (B) Proportions of reads mapping with proper pairing (Data S2, column N) against assembly of strain CVM_N17S1020. Kruskal–Wallis p = 7.956 × 10^−12^ ( df = 7) indicates all healed reads except noNmin100 and bayesHammer show an improvement proper paired mapping over raw reads by p < 0.05 under the pairwise comparison post hoc test. [file peerj-09-12446-s014.png]

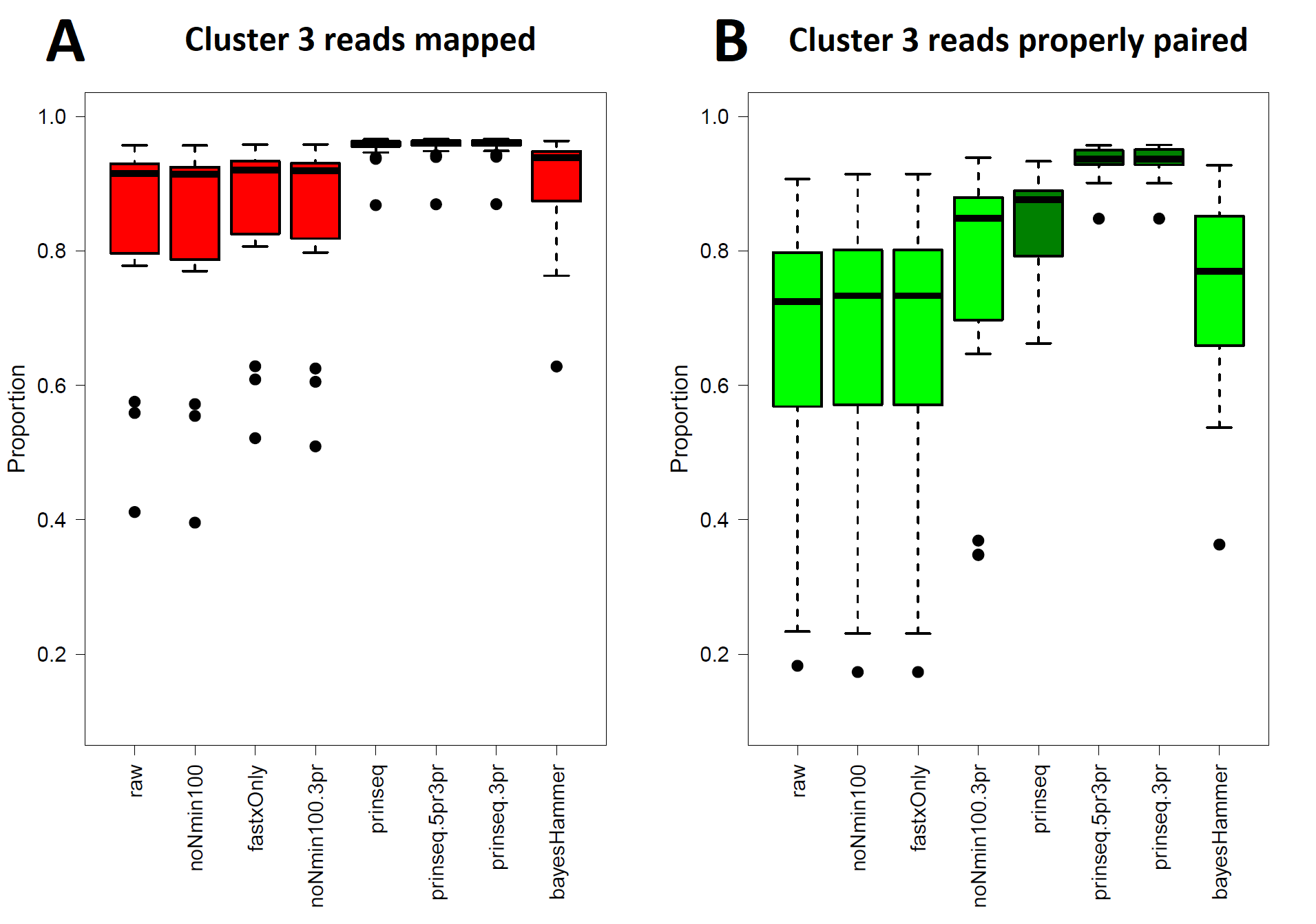

Supplement: Supplemental Information 15 — (A) Total proportions of reads mapped (Data S3, column M) to the genome of strain 2012K-0678. Kruskal–Wallis p = 2.20 × 10^16^ ( df = 7) indicates only prinseq, prinseq-5pr3pr, and prinseq-3pr read mappings differ from raw reads by p < 0.05 under the pairwise comparisons post hoc test. (B) Proportions of reads mapping with proper pairing (Data S3, column N) against the genome of strain 2012K-0678. Kruskal–Wallis p = 2.20 × 10^−16^ (df = 7) indicates only prinseq, prinseq-5pr3pr, and prinseq-3pr proper pairing rates differ from raw reads by p < 0.05 under the pairwise comparisons post hoc test. [file peerj-09-12446-s015.png]

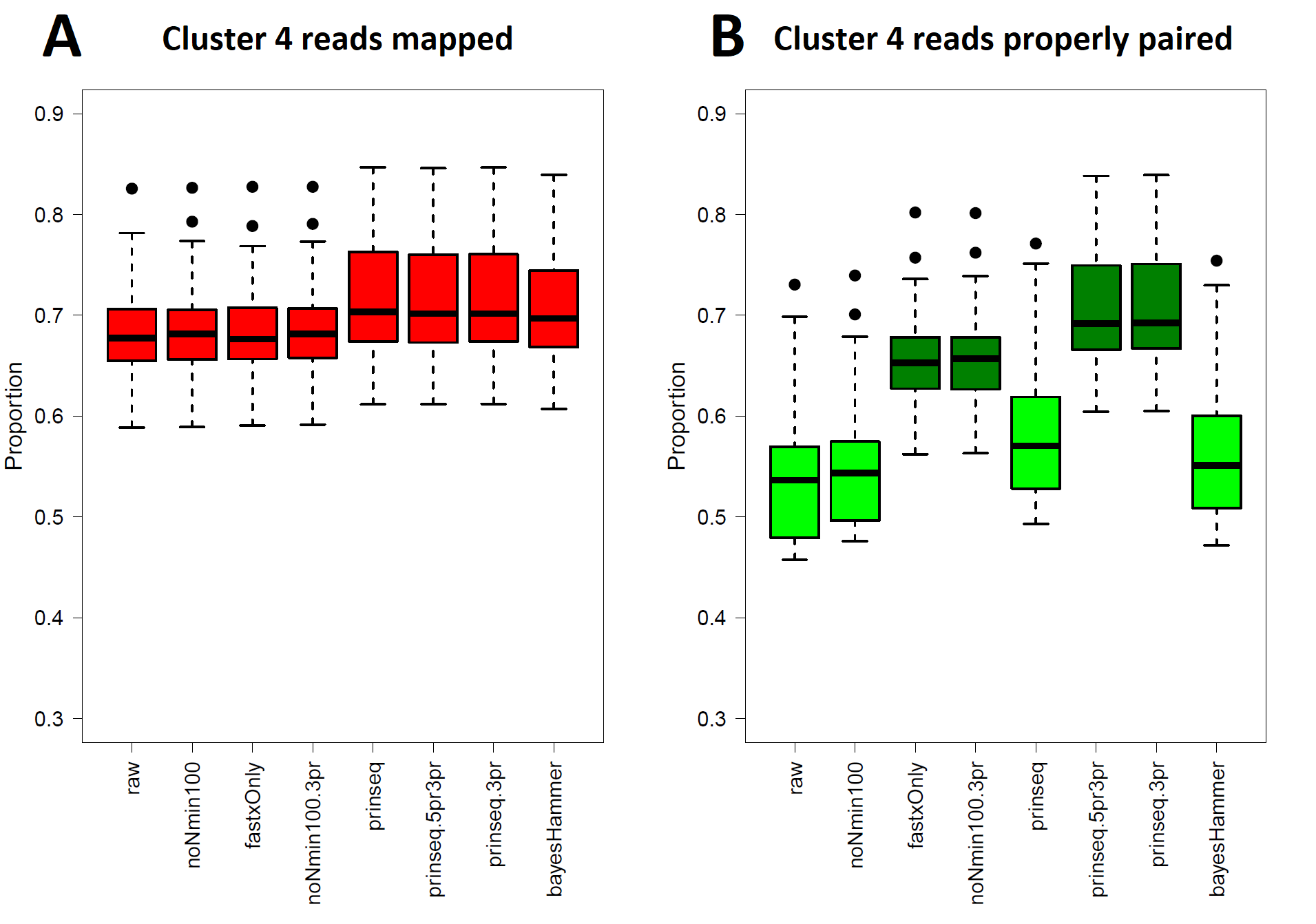

Supplement: Supplemental Information 16 — (A) Total proportions of reads mapped (Data S4, column M) to the genome of strain 2015C-3794. Kruskal–Wallis p = 0.3180 ( df = 7) indicates that none of the healing pipelines significantly improve total read mapping. (B) Proportions of reads mapping with proper pairing (Data S4, column N) against the genome, 2015C-3794. Kruskal–Wallis p = 2.7530 × 10^−13^ ( df = 7) indicates that fastxOnly-3pr, noNmin100-3pr, prinseq-5pr3pr, and prinseq-3pr have proper pairing rates above raw reads by p < 0.05 under the pairwise comparisons post hoc test. [file peerj-09-12446-s016.png]

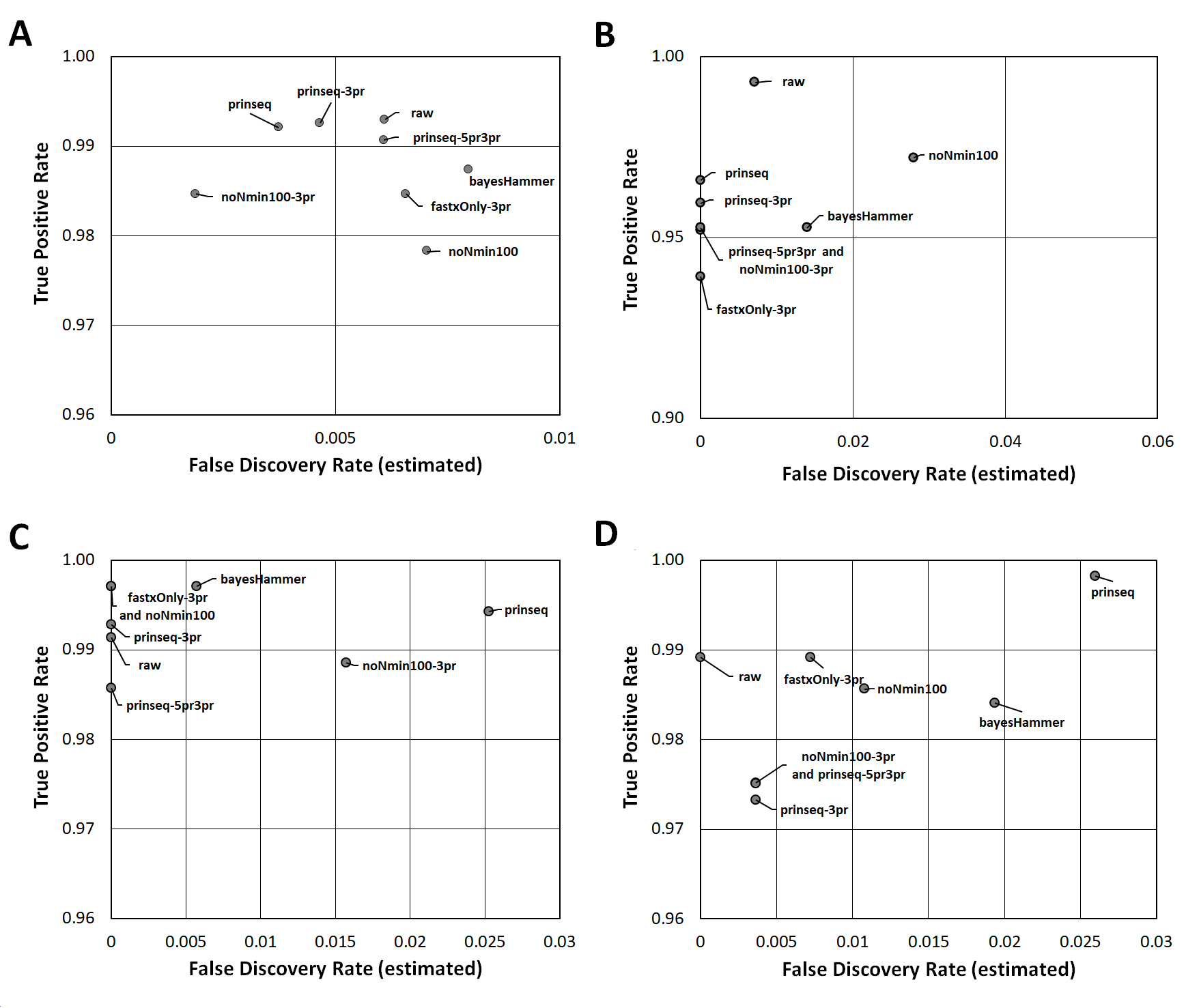

Supplement: Supplemental Information 17 — ROC-like plots of unique CFSAN SNPs (estimated false discovery rate in Data S5) compared to detected concordant SNPsor True Positive Rate(estimated sensitivity in Data S5). (A) E. coli O26 (Cluster 1). (B) S. enterica Reading (Cluster 2). (C) S. enterica Pomona(Cluster 3). (D) Shigella sonnei (Cluster 4). [file peerj-09-12446-s017.png]
